# Unraveling Sugar Chain Signatures of the “Seeds” of Tumor Metastasis

**DOI:** 10.4172/jpb.1000e31

**Published:** 2017-01-30

**Authors:** Denong Wang

**Affiliations:** Tumor Glycomics Laboratory, SRI International Biosciences Division, CA, USA

Circulating Tumor Cells (CTCs) are rare cancer cells in blood circulation that are shed from the primary tumor and play key roles in disseminating metastatic tumor cells to remote sites [1,2]. Detection of CTCs has been explored as a non-invasive “liquid biopsy” for tumor diagnosis and prognosis [3–5]. Cancer Stem Cells (CSCs) belong to a subpopulation of undifferentiated tumor cells with embryonic characteristics [6–9]. With epithelial-to-mesenchymal transition traits, CSCs are capable of escaping the primary tumor and entering the bloodstream as a subset of CTCs with high metastatic potential [9,10]. Developing targeted immunotherapy to eradicate metastatic tumor cells *in vivo* is one of the principal objectives for the next generation of precision tumor medicine.

Identifying CTC-specific cell-surface biomarkers is, however, substantially challenging. First, CTCs originate from self-epithelial cells. Biomarkers currently in use for detection and isolation of these cells, such as the cell surface marker Epithelial Cell Adhesion Molecule (EpCAM), are commonly expressed by normal epithelial cells [9,10]. The lack of specific immunological targets to detect CTCs/CSCs is a road block to development of highly specific immunotherapy against tumor metastasis. Second, CTCs/CSCs are extremely rare [11,12]. The number of CTCs detectable in blood is approximately 1 CTC per 10^6^-10^7^ of peripheral mononuclear blood cells (PBMCs). Conventional molecular and cellular techniques may not detect them and the molecular targets they express. Thus, innovative ideas and new tools are needed in the exploration of novel biomarkers for CTC detection and targeting.

An example of tools that may uncover glycan markers of CTCs is illustrated in Figure 1 [13]. In this study, glycomics tools were introduced to probe cell-surface glycan markers of breast CTCs (bCTCs). Specifically, carbohydrate microarrays were applied to screen anti-tumor antibodies to identify those that are specific for tumor glycan markers. A high-speed fiber-optic array scanning technology (FAST scan) was then applied to verify whether the identified targets are CTC-specific cell-surface markers.

In a clinical case study, blood samples from five Stage IV Metastatic Breast Cancer (MBCA) patients were characterized [13]. Glycan marker gp^C1^ positive CTCs were detected in all subjects; approximately 40% of bCTCs were strongly gp^C1^ positive. Interestingly, the CTCs from a triple-negative breast cancer (TNBC) patient with multiple sites of metastasis were predominantly gp^C1^ positive (92.5%, 37/40 CTCs). This test of basic principles demonstrates the feasibility of detecting CTC-glycan markers using FAST-scan technology.

Cell-surface expression of gp^C1^ in a panel of tumor cell lines was also characterized by a glycan-specific flow cytometry assay [13,14]. In the first set of experiments, tumor cell lines of distinct tissue origin, including a BCA line, T-47D; a Lung Cancer (LCA) line, A549; a Prostate Cancer (PCA) line, PC3; and a skin-derived melanoma cell line, SKMEL-28, were examined. SKMEL-28 (melanoma) and PC3 (PCA) were negative, A549 (LCA) was weakly positive, but T-47D (BCA) was strongly positive. In the second set of staining, a panel of seven human BCA lines was examined. These included two estrogen-receptor-positive (ER^+^) and progesterone-receptor-positive (PR^+^) lines (T-47D and MCF-7), one ER^+^ (SK-BR-3), and four triple-negative (TN) cancers that lacked the estrogen, progesterone, and Her2/neu receptors (BT-549, Hs578T, MDA-MB-231, and MDA-MB-468). T-47D and MCF-7 were strongly positive, and SK-BR-3 was intermediately positive in gp^C1^ expression. Notably, two TNBC lines, BT-549 and MDA-MB-468, were found to be strongly gp^C1^ positive. In contrast, the two remaining TNBC lines, Hs578T and MDA-MB-231, were negative.

It is noteworthy that some BCA cell lines analyzed exhibited CSC-phenotype and potency in establishing a metastatic tumor *in vivo*. For examples, the TNBC line MDA-MB-231 is phenotypically CD44^+^/CD24^−^ and is able to establish bone metastasis in nude mice. MDA-MB-468 is, however, CD44^+^/CD24^+^ and is highly efficient in lung (but not bone) metastasis [15]. MDA-MB-231 is gp^C1^-negative but the lung metastatic MDA-MB-468 and another TNBC line BT-549 are strongly positive in gp^C1^-expression [14]. These findings shed light on the glycomics diversity of metastatic tumor cells. Clearly, the repertoire of potential glycan markers of CTCs/CSCs remains largely unexplored and warrants a focused investigation.

An anti-tumor glycan monoclonal antibody (mAb) C1 [13,14] served as a key reference reagent for monitoring tumor cell-surface expression of gp^C1^. Of note, the parental hybridoma cell line of C1, called HAE3, was raised against epiglycanin, the major sialomucin glycoprotein (~500 kDa) of murine mammary adenocarcinoma TA3 cells [16–18]. However, this anti-murine carcinoma antibody exhibits strong cross-reactivity with a number of human epithelial tumors in tissues, including lung, prostate, bladder, esophageal, and ovarian cancers [17–19]. This cross-species tumor-binding profile suggests antibody recognition of a conserved tumor glycan marker that is co-expressed by both mouse- and human-derived epithelial cancers.

Carbohydrate microarrays were, therefore, introduced to explore the potential natural ligands of C1 and HAE3. For this purpose, a large collection of purified natural carbohydrate antigens was applied for the microarray screening. Importantly, a number of blood group substances were spotted in this carbohydrate microarray together with a large collection of carbohydrate antigens to critically examine the antibody binding specificity. The antibodies C1 and HAE3 selectively bonded to a number of blood group precursor antigens. These precursor substances were prepared to remove most of the α-L-fucosyl end groups that are essential for blood group A, B, H, or Lewis active side chains but possess the internal domains or core structures of blood group substances. By contrast, these mAbs had no detectable cross-reactivity with blood group substances A, B, O, or Lewis antigens, or the large panel of other carbohydrate antigens spotted in the same array.

Selective detection of these blood group precursors from a large panel of blood group substances by the mAbs illustrated they are specific for a shared cryptic glyco-epitope of these precursor substances. This microarray finding was further validated by glycan-specific enzyme-linked immunosorbent assay (ELISA) and glyco-conjugate-based epitope competition assays [13,14]. Figure 2 is a schematic of the blood group substance structure with the common blood group precursor core structure highlighted. The four types of branched structures illustrate the potential complexity of the internal portion of the carbohydrate moiety of blood group substances, which was proposed based on extensive immunochemical characterization of blood group substances [20–23].

Tumor-associated overexpression of blood-group-related autoantigens is not limited to BCA. Gao et al. recently reported that the natural ligand of a PCA-specific mAb F77 is in fact blood group H, which is built on a 6-linked branch of a poly-N-acetyllactosamine backbone [24,25]. Overexpression of gp^F77^ in PCA may reflect increased blood group H expression together with up-regulated expression of branching enzymes. HAE3 and C1 differ from F77 in glycan binding specificities and tumor-binding profiles. Unlike F77, which is blood-group-H specific and stains the PCA cell line PC3, HAE3 and C1 have neither reactivity with blood group H nor the cell surface targets of PC3. Taken together, these studies suggest epithelial tumor expression of blood group substance-related autoantigens. The potential of this class of carbohydrate-based immunological targets for tumor vaccine development and targeted immunotherapy has yet to be explored.

## Figures and Tables

**Figure 1:**
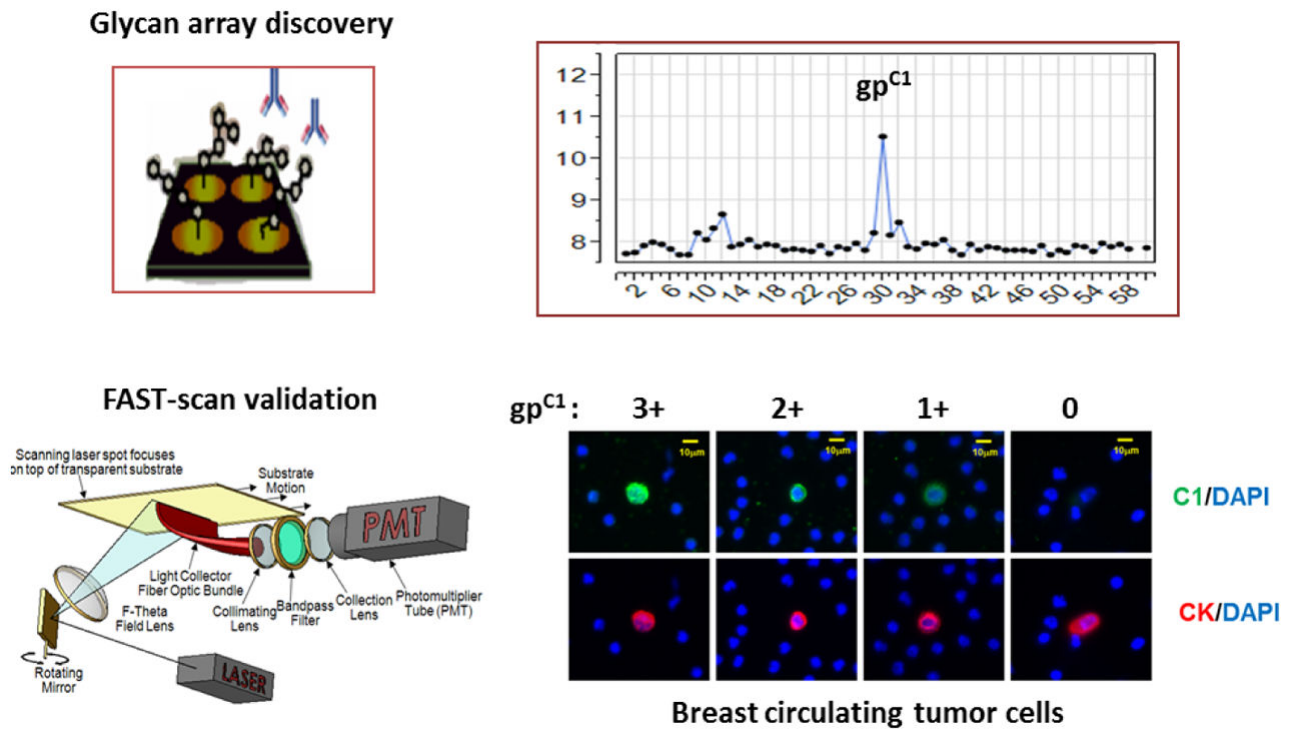
New tools to uncover glycan markers of CTCs. Top: Schematic of glycan array-based tumor biomarker discovery; Bottom: FAST-scan to explore glycan markers of CTCs. The gp^C1^ positive CTCs were stained in green in the background of the DAPI-blue labeling of white blood cells and co-stained by an anti-cytokeratin (CK) antibody in red [13].

**Figure 2:**
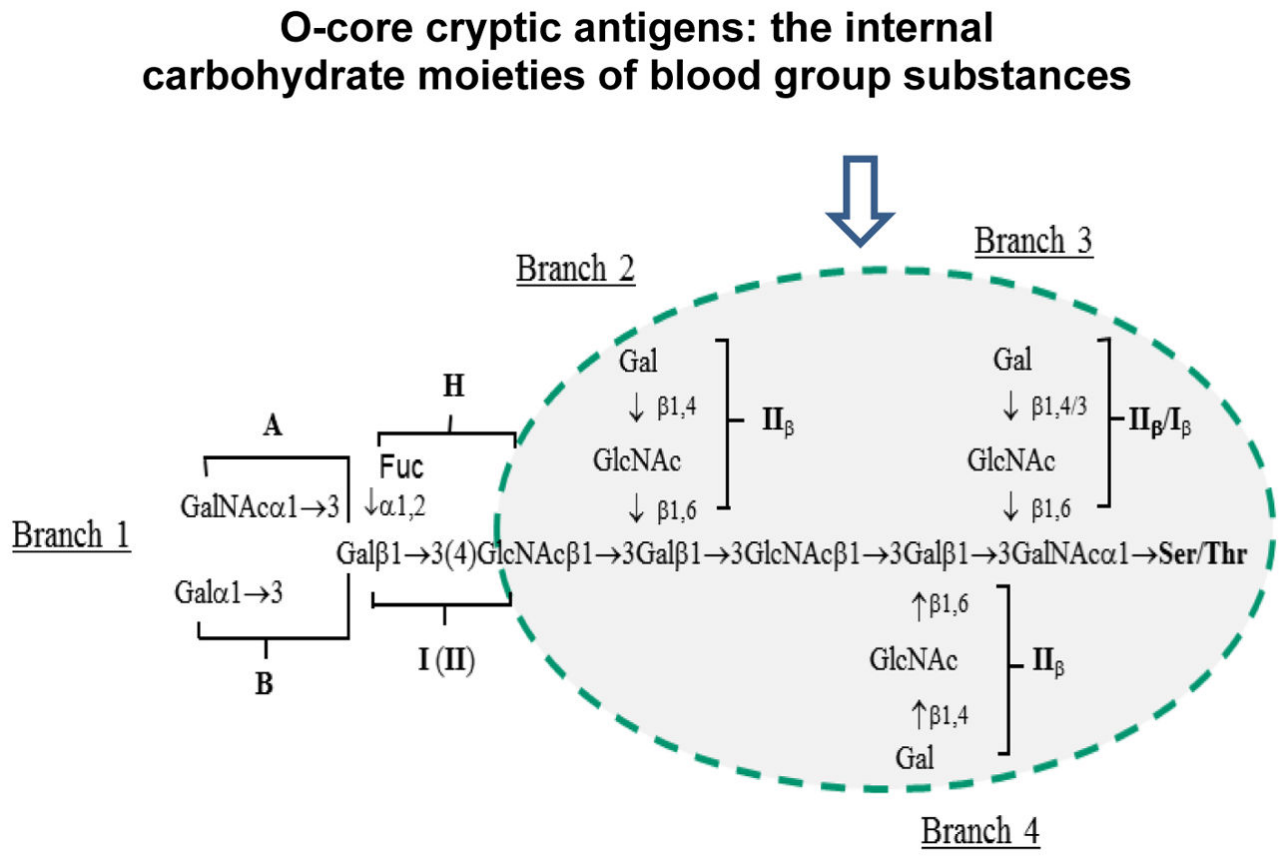
Schematic of a blood group substance structure with the conserved O-glycan core highlighted [14].
